# Molecular characterization of antifolates resistance-associated genes, *(dhfr *and *dhps) *in *Plasmodium vivax *isolates from the Middle East

**DOI:** 10.1186/1475-2875-8-20

**Published:** 2009-01-28

**Authors:** Sedigheh Zakeri, Shadi Rabiei Motmaen, Mandana Afsharpad, Navid Dinparast Djadid

**Affiliations:** 1 Malaria and Vector Research Group (MVRG), Biotechnology Research Center, Institut Pasteur of Iran, Pasteur Avenue, P.O.BOX 1316943551, Tehran, Iran; 2 Biology Department, Khatam University, Tehran, Iran

## Abstract

**Background:**

In Iran, co-infections of *Plasmodium vivax *and *Plasmodium falciparum *are common and *P. vivax *infections are often exposed to sulphadoxine-pyrimethamine (SP). In the present study, the frequency distribution of mutations associated to SP resistance was investigated in *pvdhfr *and *pvdhps *genes from field isolates.

**Methods:**

Clinical isolates of *P. vivax *were collected in two different malaria endemic regions in northern and south-eastern Iran, between 2001 and 2006. All 189 collected isolates were analysed for SNP/haplotypes at positions 13, 33, 57, 58, 61, 117 and 173 of the *pvdhfr *and 383 and 553 of *pvdhps *genes using nested PCR-RFLP methods

**Results:**

All 189 examined isolates were found to carry wild-type amino acids at positions 13, 33, 61 and 173, while 57L and 58R and 117N mutations in pure form was detected among 1.1%, 17.5% and 26% examined samples, respectively, with no polymorphisms in different loci of *dhps *genes. Based on size polymorphism of *pvdhfr *genes at repeat region, among northern isolates, the frequency distribution for type A and B were 2.2% and 97.8% respectively. However, in southern samples the prevalence of type A, B and C were 7%, 89.5% and 7.7%, respectively. Mixed genotype infections (type B and C) were detected in only 4.2% (6/143) of southern, but in none of the northern isolates. The combination of *pvdhfr and pvdhps *haplotypes among all 189 samples demonstrated six distinct haplotypes. The two most prevalent haplotypes among all examined samples were I_13_P_33_F_57_S_58_T_61_S_117_I_173_/A_383_A_553 _(65.6%) and I_13_P_33_F_57_S_58_T_61_**N**_117_I_173 _(16.4%). Two other alleles with one point mutation I_13_P_33_F_57_**R**_58_T_61_S_117_I_173_/A_383_A_553 _and two mutations I_13_P_33_F_57_**R**_58_T_61_**N**_117_I_173_/A_383_A_553 _accounted for 7.4% and 9.5% of the total isolates.

**Conclusion:**

The present molecular data provide important information for making decisions on population based drug use in Iran. In addition, since October 2005, with more availability of SP as first-line treatment, *P. vivax *isolates are more exposed to SP and the selection or spread of resistant *pvdhfr *and *pvdhps *alleles might increase in the near future in this region.

## Background

*Plasmodium vivax *remains the most widespread malaria parasite in areas outside of Africa [1] and chloroquine (CQ) remains the first-line treatment for vivax malaria in most endemic regions. In recent years, chloroquine-resistant *P. vivax *parasites have been reported in several locations, with high level resistance confirmed in parts of Thailand, Indonesia and New Guinea and in Central America [2-7]. In those countries, where sulfadoxine/pyrimethamine (SP) has been used extensively, high grade antifolate resistance has also emerged in *P. vivax *populations [8-13].

Unfortunately, resistance develops relatively quickly, when SP is widely used. Molecular and epidemiological studies of both *Plasmodium falciparum *and *P. vivax *have revealed that the dihyrofolate reductase (DHFR) and dihydropteroate synthase (DHFR) enzymes are the therapeutic targets of SP [10-17]. As a result, resistance to SP is determined by specific point mutations in the parasite *dhfr *and *dhps *genes. These mutations cause alterations of key amino acid residues in the active sites of these enzymes, which reduce the affinity of the enzyme for the drug [15,18-25]. Therefore, detection of these mutations in wild isolates has proved valuable in the mapping and monitoring of resistance for guiding malaria control measures.

Vivax infections are not often treated with SP, but *P. vivax *isolates are exposed to SP because mixed infections are common in Asia and South America [26-29] and are often mis-diagnosed. As continuous *in vitro *culture of *P. vivax *remains unavailable, and it is difficult to monitor the susceptibility of *P. vivax *to anti-malarial drugs by *in vitro *tests [30,31], the association between the various *pvdhfr *point mutations and resistance to pyrimethamine relies on epidemiological and clinical investigations. Different studies showed that in areas where there is a long history of extensive SP use, mutant alleles of *pvdhfr *gene are prevalent; however wild type *pvdhfr *has been found more commonly in areas with limited use of SP [10,12,13,17]. So far, 20 non-synonymous mutations have been described in *pvdhfr*, [31] and different studies of *P. vivax *parasites in different malaria endemic areas, such as Thailand and India, showed that mutations at *pvdhfr *codons 57, 58, 61, 117 and 173, [13,32] were found to be involved in clinical antifolate resistance [12,31]. Five mutations have already been identified in *pvdhps *gene, at codons 382, 383, 512, 553, and 585.

Iran is located in the Middle East and, in this region, *P. vivax *is the most prevalent malaria parasite species, responsible for 80 to 90% of malaria cases. The disease is endemic in the south-east bordering with Pakistan and Afghanistan, but it re-emerged in the north of the country after a 20-year interruption of transmission [33]. In Iran, CQ has been used as the first line anti-malarial treatment and SP was used as second-line treatment [34] for uncomplicated *P. falciparum *infection. Furthermore, while *in vitro *studies have reported SP resistance since 1993 [35,36], *in vivo *resistance of *P. falciparum *to SP is not yet common in these areas. With the spread of chloroquine resistance in *P. falciparum*, the Center for Diseases Management and Control (CDMC), decided in 2005 to revise its treatment policy and SP in combination with CQ has been recommended as the first-line anti-malarial treatment, with artemether-lumefantrine (Co-Artem^®^), as second-line [34]. Although SP may remain the treatment of choice for uncomplicated malaria, the high rates of treatment failures with CQ [34] and, therefore, inadequate efficacy of treatment with the SP/CQ combination, the CDMC decided in 2007 to revise its treatment policy again and SP/CQ was replaced with SP/artemisinin as the first-line recommendation for falciparum malaria. CQ remains the first choice for treatment of *P. vivax *mono-infections and resistance to either CQ or SP has not yet been recorded in Iran. In addition, in this area *P. vivax *co-exists with *P. falciparum *[26] but the correct diagnosis of mixed infections is not easy based on microscopic examination of blood films, and the clinical symptoms caused by the two species cannot be differentiated. As a result, *P. vivax *may often be treated with SP because of mixed infections and inaccurate diagnosis.

The *pvdhfr *and *pvdhps *genotype might be associated with treatment failure in individual vivax malaria patients, and while data on the genotypes of these two genes are available from Thailand, the Indian subcontinent and the Indonesian archipelago, such data are lacking in many regions, most notably Central and South America and the Middle East, with only a few isolates from those regions having been assessed for mutations in *dhfr*.

## Methods

### Study sites and *P. vivax *clinical isolates

In this study, 189 *P. vivax *clinical isolates were collected between March 2001 and March 2006, from *P. vivax*-infected patients, aged from one to > 60 years, living either in the tropical south-eastern region (Chabahar district in Sistan and Baluchistan) (n = 143), or in area of resurgent malaria (Pars Abad in Ardebil province) in the temperate northern endemic area (n = 46). The most prevalent parasite species in the temperate northern region is *P. vivax*, with *Anopheles maculipennis *and *Anopheles sacharovi *as the main vectors, and *Anopheles superpictus *and *Anopheles hyrcanus *as the secondary vectors. In the south-eastern region, transmission is year-round with two peaks, the first in May to August with *P. vivax *as the predominant species and the second peak from October to November when both *P. falciparum *and *P. vivax *infections are equally recorded. The main mosquito vectors in the south-eastern region are *Anopheles stephensi*, *Anopheles culicifacies*, *Anopheles fluviatilis *and *Anopheles pulcherrimus*.

All subjects had slide and PCR-proven infection by *P. vivax*. None of the subjects received SP therapy and infections were treated with chloroquine. In both study areas the patients have access to anti-malarial drugs through local health centers.

Informed consent was obtained from patients or parents of patients before inclusion in the study. The study was reviewed by, and received Ethical Clearance from Institut Pasteur of Iran. Blood samples were collected in tubes containing EDTA, stored at 4°C and then transported to the main laboratory in Tehran.

### Parasite genomic DNA extraction

Parasite DNA was extracted from 250 μl infected whole blood by phenol/phenol-chloroform extraction and ethanol precipitation as described previously [37]. The DNA was dissolved in 30 μl TE buffer (10 mM Tris-HCL pH 8.0, 0.1 mM EDTA). For the detection of point mutations at residues 13, 33, 57, 58, 61, 117 and 173, the previously described PCR-RFLP protocols were used with some modifications [12,13,38].

### Primary amplification of *pvdhfr*

In the first reaction, the entire *P. vivax dhfr-ts *gene was amplified by the primers VDT-OF: ATGGAGGACCTTTCAGATGTATTTGACATT and VDT-OR: GGCGGCCATCTCCATGGTTATTTTATCGTG [13]. The cycling conditions for the nest-1 reaction was as follows: 95°C for 5 min, 25 cycles of 64°C for 2 min, 72°C for 2 min, 94°C for 1 min followed by 64°C for 2 min and 72°C for 15 min.

#### PCR amplification of positions 13, 33, 58, and 61

One μl product of the first reaction was then used in second round of amplification using the following primers for positions 13, 33, 58, and 61:

VDFN13F: GACCTTTCAGATGTATTTGACATTTACGGC

VDFN13R: GGTACCTCTCCCTCTTCCACTTTAGCTTCT

The cycling conditions for the nest-2 reaction was as follows: 95°C for 5 min, 25 cycles of 66°C for 2 min, 72°C for 2 min, 94°C for 1 min followed by 66°C for 2 min and 72°C for 15 min.

#### RFLP

To detect mutation at position 13L, 10 μl of the PCR products were digested with 10 U *HaeIII *enzyme (Roche, Germany) for 1 h at 37°C in a total volume of 20 μl (232 bp = 32 bp + 200 bp). To detect mutations at residue P33L and S58R, 10 μl of the PCR products were digested with 10 U *Cfr42I (SacII) *(Fermentase, Vilnius, Lithuania) and *AluI *(Roche, Germany) for 4 h at 37°C in a total volume of 20 μl (232 bp = 94 bp + 138 bp for P33 and 232 bp = 25 bp + 207 bp for 58R, 232 bp = 25 bp+40 bp+167 bp for wild type S58), respectively. Mutation at residue 61 M was detected by digestion of 10 μl of the PCR products with 10 U *Tsp45I *(New England Biolab, Beverly, MA, USA) for 4 h at 37°C in a total volume of 20 μl (232 bp = 32 bp + 200 bp).

#### PCR amplification of positions 57 and 173

The product of first reaction was also amplified with oligonucleotide pair:

VDT-OF: ATGGAGGACCTTTCAGATGTATTTGACATT

VDFNR: TCACACGGGTAGGCGCCGTTGATCCTCGTG

to amplify positions 57 and 173. The cycling conditions for the nest-2 reaction was as follows: 95°C for 5 min, 25 cycles of 66°C for 2 min, 72°C for 2 min, 94°C for 1 min followed by 66°C for 2 min and 72°C for 15 min. It should be noted that because of presence of repeat regions that amplify the above-mentioned primers, there are size variations among the amplified PCR products, which affect the size of digested products. In the present study, the size of the digested PCR products, which is reported in the following RFLP section, is based on *pvdhfr *Accession no: X98123.

#### RFLP

To detect mutation at residue F57, 10 μl of the PCR products were digested with 10 U *XmnI: *(New England Biolab, Beverly, MA, USA) for 4 h at 37°C in a total volume of 20 μl (608 bp = 166 bp + 442 bp). Mutation at residue I173L was detected by digestion of 10 μl of the PCR products with 10 U *Eco130I (StyI) *(Fermentase, Vilnius, Lithuania) for 4 h at 37°C in a total volume of 20 μl (wild type I173: 608 bp = 136 bp + 472 bp and mutant type 173L: 608 bp = 73 bp + 97 bp + 438 bp).

#### PCR amplification of positions 57 and 117

One μl product of first reaction was also amplified with primers:

VDNF57:CATGGAAATGCAACTCCGTCGATATGATGT

VDF-NR:TCACACGGGTAGGCGCCGTTGATCCTCGTG

The cycling conditions for the this reaction was 95°C for 5 min, 25 cycles of 66°C for 2 min, 72°C for 2 min, 94°C for 1 min followed by 66°C for 2 min and 72°C for 15 min.

#### RFLP

To detect mutation at residue 57I, 10 μl of the PCR products were digested with 10 U BsrGI (New England Biolab, Beverly, MA, USA) for 4 h at 37°C in a total volume of 20 μl (472 bp = 28 bp + 444 bp). To detect mutation at residue S117N/T, 10 μl of the PCR products (472 bp) were digested with 10 U *PvuII *(New England Biolab, Beverly, MA, USA) (S117, 214 bp + 258 bp), for 4 h at 37°C and *BsrI *(New England Biolab, Beverly, MA, USA (117N, 219 bp + 253 bp), *BstNI *(New England Biolab, Beverly, MA, USA) (117T, 215 bp + 257 bp) for 1 h at 65°C in a total volume of 20 μl. All amplifications were carried out in a final volume of 25 μl, which included 1 μl of template from either genomic DNA or the primary reaction. The primers were used at a final concentration of 250 nM and the reaction mixture contained 10 mM Tris-HCL (pH 8.3), 50 mM KCl, 2 mM MgCl_2_, each of the four deoxynucleotide triphosphates at a concentration of 125 μM, and 0.2 U of Taq polymerase (Invitrogen, Carlsbad, CA). The DNA fragments obtained following PCR amplification or RFLP analysis were electrophoresed on 2.5% (Invitrogen, Carlsbad, CA) and 3% Metaphor (Invitrogen, Carlsbad, CA) agarose gels, respectively. All digested products of RFLP were subjected to electrophoresis on 3% MetaPhor agarose gels.

#### Analysis of *pvdhfr *gene at repeat region

This region was amplified using 1 μl of primary reaction with primers:

VDFN2F: CGGTGACGACCTACGTGGATGAGTCAAAGT

VDFN2R: TAGCGTCTTGGAAAGCACGACGTTGATTCT as described previously [12]. The cycling conditions for the this reaction was 95°C for 5 min, 25 cycles of 66°C for 2 min, 72°C for 2 min, 94°C for 1 min followed by 66°C for 2 min and 72°C for 15 min. The DNA fragments obtained following PCR amplification were analysed following electrophoresis on 3% Metaphor agarose gels.

### Amplification of *pvdhps*

The *pvdhps *gene was amplified from genomic DNA by nested PCR. The primers used in the first and second round PCRs were described previously [38]. In the first reaction *pvdhps *was amplified with primers:

VDHPS-OF: ATTCCAGAGTATAAGCACAGCACATTTGAG

VDHPS-OR: CTAAGGTTGATGTATCCTTGTGAGCACATC

The second amplification was performed with the primers for detection of 383 mutation:

VDHPS-NF: AATGGCAAGTGATGGGGCGAGCGTGATTGA

VDHPS-NR: CAGTCTGCACTCCCCGATGGCCGCGCCACC

for detection of the 553 mutations the oligonuclotide primers

VDHPS-553OF: TTCTCTTTGATGTCGGCCTGGGGTTGGCCA

VDHPS-NR: CAGTCTGCACTCCCCGATGGCCGCGCCACC were used.

The cycling conditions for the nest-1 PCR reaction was as follows: 95°C for 5 min, 25 cycles of 58°C for 2 min, 72°C for 2 min, 94°C for 1 min followed by 58°C for 2 min and 72°C for 15 min and for nest-2 PCR was: 95°C for 5 min, 25 cycles of 50°C for 2 min, 72°C for 2 min, 94°C for 1 min followed by 50°C (for VDHPS-553OF and VDHPS-NR 50°C) for 2 min and 72°C for 15 min.

#### RFLP

To detect mutation at residue A383G, 10 μl of the PCR products were digested with 10 U *MspI (HpaII) *(Fermentase, Vilnius, Lithuania) (mutant 383G, 703 bp = 48 bp + 655 bp) and *MscI *(New England Biolab, Beverly, MA, USA) (wild A553, 170 bp = 27 bp + 143 bp) for 4 h at 37°C in a total volume of 20 μl.

## Results

### Distribution of mutations in *pvdhfr *and *pvdhps*

All 189 isolates from north and south were analysed for SNP/haplotypes at positions 13, 33, 57, 58, 61, 117 and 173 of the *pvdhfr *and 383 and 553 of *pvdhps *genes using PCR-RFLP methods [12,13,38]. In *pvdhfr*, polymorphisms at positions 57L, 58R and 117N have been found in 1.4%, 21.7% and 30% of southern isolates, respectively (Table 1). Among northern isolates mutations at 58R and 117N were found in 4.4% and 13% of the studied isolates, respectively (Table 1).

**Table 1:** The frequency distribution of SNPs combinations of *pvdhfr *and *pvdhps *alleles associated with sulphadoxine/pyrimethamine in *Plasmodium vivax *isolates from north and southeastern, Iran

***Pvdhps***		***pvdhfr***									
**A383G**	**A553G**	**I13L**	**P33L**	**F57I/L**	**S58R**	**T61M**	**S117T/N**	**I173L**	**South (%)** **n = 143**	**North (%)** **n = 46**	**Total (%)** **n = 189**

A	A	I	P	F	S	T	S	I	85 (59.4%)	39 (84.8%)	124 (65.6%)

A	A	I	P	F	S	T	**N**	I	26 (18.2%)	5 (10.8%)	31 (16.4%)

A	A	I	P	F	**R**	T	S	I	13 (9.1%)	1 (2.2%)	14 (7.4%)

A	A	I	P	F	**R**	T	**N**	I	17 (11.9%)	1 (2.2%)	18 (9.6%)

A	A	I	P	**L**	S	T	S	I	1 (0.7%)	-	1 (0.5%)

A	A	I	P	**L**	**R**	T	S	I	1 (0.7%)	-	1 (0.5%)

-	-	-	-	**L = 1.1%**	**R = 17.5%**	-	**N = 26%**	-			

In total, all 189 examined isolates were found to carry wild-type amino acids at positions 13, 33, 61 and 173, while 57L and 58R and 117N mutations in pure form was detected among 1.1%, 17.5% and 26% examined samples, respectively (Table 1). In the case of *pvdhps *gene, polymorphisms in different loci of *dhps *(A383G and A553G) were investigated and no mutations were detected at all in the examined samples.

### Size polymorphism of *pvdhfr *at repeat region

In this investigation all three types A, B and C [12] were found among southern isolates, but only types A and B were detected among northern isolates. The frequency distribution for type A and B were 2.2% (1/46) and 97.8% (45/46) among northern isolates, respectively. However, for southern samples the prevalence of the type A, B and C were 7%, 89.5% and 7.7%, respectively. Mixed genotype infections (type B and C) were detected in only 4.2% (6/143) of southern, but not in northern isolates.

### Distribution of *pvdhfr *and *pvdhps *haplotypes in Iran

**Figure 1:**
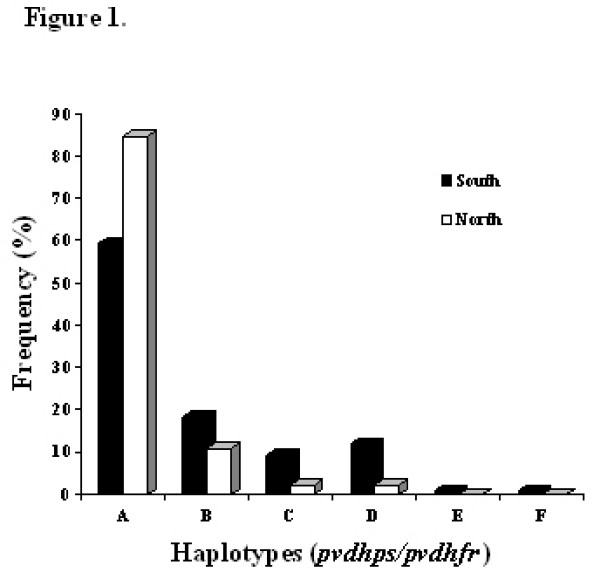
**Frequency distribution of the combination *pvdhfr/pvdhps *haplotypes obtained from 189 isolates collected in Ardebil province in north and Sistan and Baluchistan province in southeastern of Iran**. The haplotype I_13_P_33_F_57_S_58_T_61_S_117_I_173_/A_383_A_553 _was the most prevalent among northern (84.8%) and southern (59.4%) *P. vivax *isolates. All six haplotypes are indicated as A to F in the figure. Mutated amino acids are boldfaced. A) I_13_P_33_F_57_S_58_T_61_S_117_I_173_/A_383_A_553_. B) I_13_P_33_F_57_S_58_T_61_**N**_117_I_173_/A_383_A_553_. C) I_13_P_33_F_57_**R**_58_T_61_S_117_I_173_/A_383_A_553_. D) I_13_P_33_F_57_**R**_58_T_61_**N**_117_I_173_/A_383_A_553_. E) I_13_P_33_**L**_57_S_58_T_61_S_117_I_173_/A_383_A_553_. F) I_13_P_33_**L**_57_**R**_58_T_61_S_117_I_173_/A_383_A_553_.

The combination of *pvdhfr and pvdhps *haplotypes among all 189 samples in this study demonstrated six distinct haplotypes (Figure 1). The two most prevalent haplotypes among all examined samples were I_13_P_33_F_57_S_58_T_61_S_117_I_173_/A_383_A_553 _(65.6%) and I_13_P_33_F_57_S_58_T_61_**N**_117_I_173_/A_383_A_553 _(16.4%). Two other alleles with one point mutation at position I_13_P_33_F_57_**R**_58_T_61_S_117_I_173_/A_383_A_553 _and two mutations at position 58R and 117N (I_13_P_33_F_57_**R**_58_T_61_**N**_117_I_173_/A_383_A_553_) accounted for 7.4% and 9.5% of the total isolates. This double mutant haplotype was the most frequently mutated haplotype observed among Iranian samples. Regarding these nine SNPs in *pvdhfr *and *pvdhps *genes, significant increasing in the prevalence of double mutated haplotypes (I_13_P_33_F_57_**R**_58_T_61_**N**_117_I_173_/A_383_A_553_) was observed in collected samples in year 2006 compare to 2001 (P > 0.005) (Table 2). In addition, the majority of isolates from north and also south were wild haplotype and I_13_P_33_**L**_57_**R**_58_T_61_S_117_I_173_/A_383_A_553 _(0.7%) mutant haplotype was only detected among southern but northern isolates (Table 1).

**Table 2:** Distribution of *dhfr *and *dhps *polymorphisms of *P. vivax *in 143 samples collected in 2001 to 2006 from south-eastern of Iran

***pvdhps/pvdhfr *Haplotype**	**2001**	**2003**	**2004**	**2005**	**2006**
**n = 143**	**n = 36**	**n = 16**	**n = 33**	**n = 29**	**n = 29**
**AAIPFSTSI**	30 (83.33)	9 (56.25%)	19 (57.58%)	14 (48.28%)	13 (44.83%)

**AAIPFSTNI**	2 (5.56%)	5 (31.25%)	6 (18.18%)	8 (27.59%)	5 (17.24%)

**AAIPFRTSI**	3 (8.33%)	1 (6.25%)	3 (9.09%)	4 (13.79%)	2 (6.89%)

**AAIPFRTNI**	1 (2.78%)	1 (6.25%)	4 (12.12%)	3 (10.34%)	8 (27.59%)

**AAIPLSTSI**	-	-	1 (3.03%)	-	-

**AAIPLRTSI**	-	-	-	-	1 (3.45%)

## Discussion

In regions where *P. falciparum *and *P. vivax *co-exist, it is crucial to identify effective treatment regimens that work against both parasite species. In malaria-endemic areas co-infection of *P. vivax *and *P. falciparum *is common and the long history of SP use has exposed *P. vivax *to this drug for decades. In the present study, the SP resistance-associated genes, *pvdhfr *and *pvdhps*, were analysed in samples collected (prior to introduction of SP as first-line anti-malarial), from re-emerged area in north and endemic region in south where both CQ and SP (in combination with primaquine) were used for treatment. In the south, although CQ still remains effective against *P. vivax *infection, the *in vivo *work in 2005 [39] showed that parasite clearance time increased compared to 2001 in Sistan and Baluchistan province, indicating that this could be an early sign of reduced susceptibility of the parasites to CQ. Therefore, effective alternative drug against *P. vivax *resistance to CQ might be needed. In this investigation, four and six distinct haplotypes of *pvdhfr *and only wildtype of *pvdhps *were detected among northern and southern isolates, respectively. The double mutant I_13_P_33_F_57_**R**_58_T_61_**N**_117_I_173_/A_383_A_553 _was the second frequent haplotype in our examined isolates. This is the first time that mutations at associated genes to SP resistance have been described in a large number of *P. vivax *isolates from the Middle East.

Mutations in *pvdhfr*, including 58R and 117N, have been implicated in *in vivo *pyrimethamine resistance and seem to arise first under drug pressure. The 58R was found in 17.5% of all examined isolates alone and in combination with 117N in 9.5%, despite the fact that SP had never been used as a first-line treatment for falciparum malaria before October 2005. The work carried out by Tahar and colleagues [40] showed that the 58R/117N mutant had a lower affinity for pyrimethamine and cycloguanil than did the wild type enzyme. In addition, Leartsakulpanich *et al *screened pyrimethamine resistance-associated genes and found that the 117N, 58R/117N, 58R and 173L mutant enzymes were more resistance to this drug than the wild type [41]. The similar work by Hastings and colleagues confirmed these results [11]. Regarding the clinical efficacy of SP against *P. vivax*, several workers concluded that the clinical response to SP depends on *pvdhfr *and *pvdhps *genotype [11,12,17]. They also showed that those patients who harboured triple and quadruple mutant parasites (57L/58R/61M/117T) compared with those who harboured wild type parasites were significantly associated more likely to SP treatment failure [11,12,17]. In addition, treatment failure was more frequently associated with multiple mutations in *dhfr *and *dhps *[38]. This correlation between two genes also reported for *P. falciparum*, as parasite carried wild type alleles of *dhfr *and *dhps*, the patient is likely to have an adequate clinical response to SP, but when the parasite carries mutant alleles of both genes, clinical effectiveness is compromised [42-48]. Although none of the *P. vivax *isolates have been tested in this study for their clinical response to SP, triple and quadruple mutant types, found in Thailand, India, and in Indonesia have previously been shown to be associated with a high risk of SP treatment failure [11,12,17].

The frequency distribution of *pvdhfr *mutant haplotypes was significantly higher in the endemic southern regions than re-emerged northern region (Pars-Abad, Ardebile). This might be caused by the level of disease endemicity in the south and longer usage of SP for treatment of *P. falciparum *infections. In other words, SP never used in north for treatment of malaria disease as any *P. falciparum *infections was not detected by microscopy method; therefore no SP pressure might be responsible for prevalence of mutant alleles of *dhfr *in this region. In fact, the limited diversity of *pvdhfr *mutant, particularly double mutants in northern compare to southern isolates may also be an indication for a founder effect linked to the introduction of malaria from Azerbaijan and Armenia to northern part of Iran. The presence of I_13_P_33_**L**_57_**R**_58_T_61_ S_117_I_173_/A_383_A_553 _haplotype only among southern isolates may be related to human migration between Iran and Pakistan, resulting to introduction of such haplotypes from the Indian subcontinent, where it is prevalent [31].

In the present study, the most common haplotypes of *pvdhfr *were wild type and double mutant (58R and 117N); but triple and quadruple mutants were not detected among examined isolates. In contrast, molecular analysis of *pvdhfr *among Indian field isolates showed 14 haplotypes from wild type to quadruple mutant genotype, and wild type and double mutant were still the most common [31]. Since 2005, based on the national drug policy, the anti-malarial treatment in Iran has changed and the SP became the first choice drug for treatment. With more availability of SP, there is a risk of a changing pattern of resistance of both *P. falciparum *and *P. vivax *to SP, as this closely follows the intensity with which SP has been used [31]. In addition, other antifolates such as co-trimoxazole that are routinely used against urinary tract infections and chronic bronchitis in the region could add to the overall antifolate pressure in Iran.

## Conclusion

The present molecular data provide important information for making decisions on population based drug use in Iran. In addition, previous study showed that in regions where the wild type or single mutated *pvdhfr *alleles are prevalent, SP could be a useful therapy [31] for the asexual erythrocytic stages of vivax in areas where CQ treatment failure has been reported.

## Competing interests

The authors declare that they have no competing interests.

## Authors' contributions

SZ designed and supervised the study, analysed the data and wrote the manuscript. SRM contributed in the laboratory work and MA contributed in the laboratory work and helped with analysis of the data. NDD helped with the preliminary analysis of the data and also critical reading of the manuscript.
